# Looking beneath the surface: the importance of subcortical structures in frontotemporal dementia

**DOI:** 10.1093/braincomms/fcab158

**Published:** 2021-07-16

**Authors:** Martina Bocchetta, Maura Malpetti, Emily G Todd, James B Rowe, Jonathan D Rohrer

**Affiliations:** Dementia Research Centre, Department of Neurodegenerative Disease, UCL Queen Square Institute of Neurology, University College London, London, UK; Department of Clinical Neurosciences and Cambridge University Hospitals NHS Trust, University of Cambridge, Cambridge, UK; Dementia Research Centre, Department of Neurodegenerative Disease, UCL Queen Square Institute of Neurology, University College London, London, UK; Department of Clinical Neurosciences and Cambridge University Hospitals NHS Trust, University of Cambridge, Cambridge, UK; Medical Research Council Cognition and Brain Sciences Unit, University of Cambridge, Cambridge, UK; Dementia Research Centre, Department of Neurodegenerative Disease, UCL Queen Square Institute of Neurology, University College London, London, UK

**Keywords:** frontotemporal dementia, MR imaging, subcortical structures

## Abstract

Whilst initial anatomical studies of frontotemporal dementia focussed on cortical involvement, the relevance of subcortical structures to the pathophysiology of frontotemporal dementia has been increasingly recognized over recent years. Key structures affected include the caudate, putamen, nucleus accumbens, and globus pallidus within the basal ganglia, the hippocampus and amygdala within the medial temporal lobe, the basal forebrain, and the diencephalon structures of the thalamus, hypothalamus and habenula. At the most posterior aspect of the brain, focal involvement of brainstem and cerebellum has recently also been shown in certain subtypes of frontotemporal dementia. Many of the neuroimaging studies on subcortical structures in frontotemporal dementia have been performed in clinically defined sporadic cases. However, investigations of genetically- and pathologically-confirmed forms of frontotemporal dementia are increasingly common and provide molecular specificity to the changes observed. Furthermore, detailed analyses of sub-nuclei and subregions within each subcortical structure are being added to the literature, allowing refinement of the patterns of subcortical involvement. This review focuses on the existing literature on structural imaging and neuropathological studies of subcortical anatomy across the spectrum of frontotemporal dementia, along with investigations of brain–behaviour correlates that examine the cognitive sequelae of specific subcortical involvement: it aims to ‘look beneath the surface’ and summarize the patterns of subcortical involvement have been described in frontotemporal dementia.

## Teaser

Bocchetta et al. provide an overview of the subcortical structure involvement in the clinical, genetic and pathological forms of frontotemporal dementia. Their anatomy is described with *in vivo* imaging and *post mortem* studies to discuss how neurodegenerative processes in these structures can lead to the heterogenous symptoms of frontotemporal dementia.

## Graphical Abstract

**Graphical Abstract:**
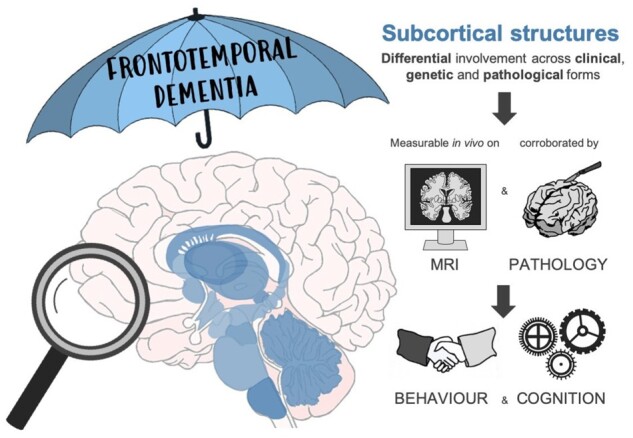

## Introduction

Frontotemporal dementia (FTD) is a common cause of early onset dementia, approximately equal in frequency to Alzheimer’s disease in people under the age of 65. It is clinically heterogeneous with symptoms, including behavioural, language, cognitive and motor deficits. Behavioural variant FTD (bvFTD) is the most common presentation, with impaired social conduct and personality changes,^1^ whilst less frequently, people present with progressive decline in speech and language functions [primary progressive aphasia (PPA)], of which there are multiple variants: semantic variant (svPPA), non-fluent variant (nfvPPA) and logopenic variant (lvPPA).^2^ People on this spectrum can also develop motor features consistent with either amyotrophic lateral sclerosis (ALS) or parkinsonism [including progressive supranuclear palsy (PSP), or corticobasal syndrome (CBS)].^3^ At present the only known risk factors for FTD are age and genetics: about a third of cases are due to an autosomal dominant mutation in microtubule-associated protein tau (*MAPT*), progranulin (*GRN*) or chromosome 9 open reading frame 72 (*C9orf72*) genes.^4^ Pathologically, three major groups are described according to the main abnormal protein seen in neuronal or glial inclusions— tau, TAR DNA-binding protein 43 (TDP-43) and fused-in-sarcoma (FUS)^5^^,^^6^— with multiple subtypes seen within each main group.

Anatomically, FTD has traditionally been characterized as a cortical dementia with atrophy predominantly of the frontal and temporal lobes, hence its name. However, imaging and neuropathological studies have identified not only other cortical areas (including the insula and anterior cingulate) but also subcortical structures as key areas of FTD-related degeneration,^7^ even at the very early stages of the disorder^8–16^ and presymptomatic phases.^17^

Behavioural studies have highlighted the relevance of subcortical structures in the development of the typical symptoms of FTD. Subcortical structures contribute to functional and structural brain networks that are affected in FTD. For example, a reward network related to the limbic system^18^ regulates appropriate behaviour for a given context by the evaluation of motivational and emotional content of the stimuli. Abnormal functioning of this circuit in bvFTD leads to abnormal responses to rewards (including food, sex and substance use).^19^

**Figure 1:**
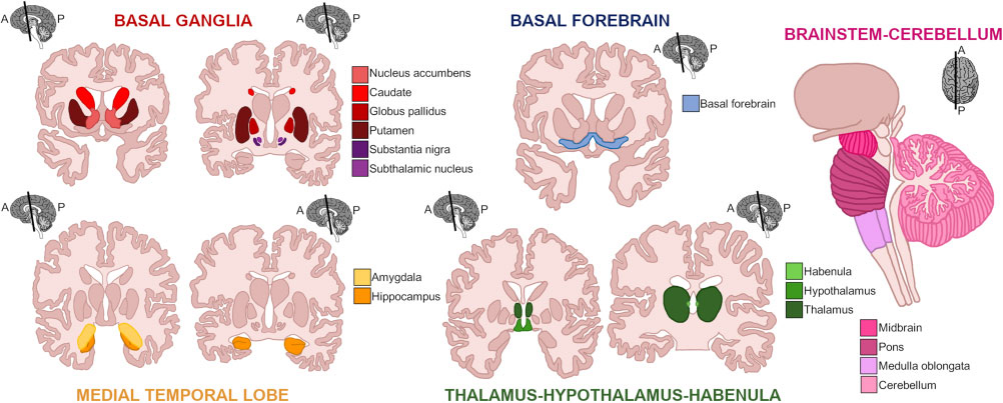
Subcortical structures involved in frontotemporal dementia. Structures are grouped and coloured based on their location and anatomical organization. The basal ganglia include the striatum (nucleus accumbens, caudate and putamen), the globus pallidus, substantia nigra and subthalamic nucleus. The amygdala and hippocampus are located in the medial temporal lobe, while the thalamus, hypothalamus and habenula are part of the diencephalon. Below the cerebrum, lie the cerebellum and brainstem (midbrain, pons and medulla oblongata).

This review aims to provide a comprehensive overview of the involvement of subcortical structures in the FTD spectrum (Fig. 1), identified by structural magnetic resonance (MR) imaging, with neuropathological corroboration of the imaging data.

For each of the structures, we first describe their anatomy and structural connections (‘Anatomy’), and then report the MRI studies that have investigated changes *in vivo* in their volume or morphology within the genetic, clinical and pathological forms of FTD (‘Neuroimaging’). This is followed by the description of which studies have reported abnormal findings at *post mortem* examination (‘Neuropathology’), and then finally, in the ‘Symptomatology’ section, we discuss how such structural changes contribute to the behavioural and cognitive deficits seen in people with FTD.

### Basal ganglia

#### Striatum

##### Anatomy

The striatum consists of dorsal and ventral regions. The dorsal striatum comprises the caudate and putamen and is primarily associated with sensorimotor functions, whilst the ventral striatum includes the nucleus accumbens and is a component of the limbic circuit, which modulates behaviour and memory.^20^

The striatum has multiple parallel connections with the prefrontal cortex, anterior cingulate cortex, orbitofrontal cortex, insula, inferior and middle temporal gyrus, and thalamus.^21^ Anatomical and physiological studies have identified functionally distinct but anatomically analogous cortico-striato-pallido-thalamic-cortical circuits. These process information in parallel and have separate connections and functions.^20^^,^^21^ In the *motor circuit* and *oculomotor circuit*, the putamen and the caudate receive input from the primary motor, somatosensory, premotor, supplementary motor and posterior parietal cortex, together with the frontal eye fields. They then send their output to the supplementary motor cortex and frontal eye fields, via the globus pallidus (internal segment), substantia nigra (pars reticulata) and thalamus (ventrolateral and ventral anterior and mediodorsal nuclei).^20^^,^^22^ The other three circuits originate from and end in the frontal cortex, but they have different pathways and have different roles in cognition, emotion and motivation.^21^ The *dorsolateral prefrontal circuit* is associated with executive function. The dorsolateral prefrontal cortex, together with the posterior parietal and premotor cortex, is connected to the dorsolateral head of the caudate, to the globus pallidus and substantia nigra, and to the thalamus (ventral anterior and mediodorsal nuclei). The *lateral orbitofrontal circuit* regulates inhibition and impulses. It is connected to the ventral anterior and mediodorsal nuclei of the thalamus via the ventromedial head of the caudate (which also receives inputs from the superior temporal gyrus, inferior temporal gyrus and anterior cingulate) and the globus pallidus and substantia nigra. In the *anterior cingulate circuit*, the nucleus accumbens and ventromedial caudate receive input from the anterior cingulate, and limbic and paralimbic regions (hippocampus, entorhinal cortex, insula, amygdala, superior and inferior temporal gyrus and temporal pole) with output via the globus pallidus and substantia nigra to the mediodorsal thalamus. This is the crucial pathway controlling motivation.^20^^,^^21^

The nucleus accumbens additionally projects to the basal forebrain and the lateral preoptic area and lateral hypothalamus.^23^ This nucleus can be further divided into a ‘shell’ and ‘core’, at least in preclinical models: the shell is connected to the medio-temporal regions (hippocampal cornu ammonis 1—CA1, CA3 and subiculum, entorhinal cortex, basolateral amygdaloid nucleus), the paraventricular thalamic nucleus and the caudal brainstem, while the functionally distinct core is connected to the dorsomedial prefrontal cortex, anterior cingulate, insula, parahippocampal cortex, midline and intralaminar thalamic nuclei, and the basolateral amygdaloid nucleus.

##### Neuroimaging

Clinically, the caudate is affected in both behavioural and language phenotypes of FTD. Compared to controls, the caudate is 11–25% smaller in bvFTD, 21% smaller in nfvPPA (worse on the left) and 8% smaller in svPPA (worse on the left).^7^^,^^14^^,^^16^^,^^24–29^ Similarly, the putamen is affected across all clinical syndromes being 7–28% smaller in bvFTD, 13% smaller in nfvPPA (worse on the left) and 11–21% smaller in svPPA (worse on the left) than controls.^7^^,^^14^^,^^16^^,^^26^^,^^29^^,^^30^ The subregions of the putamen may be equally affected by each syndrome.^25^

Among the genetic forms of FTD, *GRN* mutation carriers in particular have shown involvement of the dorsal striatum.^17^^,^^31^ Patients with *GRN* mutations show especially severe atrophy in the caudate (56% reduction versus controls), while putamen atrophy is similar to sporadic cases (27% reduction versus controls).^32^ A recent study in a large cohort of mutation carriers has found that the *GRN* group showed smaller volumes in the putamen (17% difference versus controls) and caudate (5%) only when they were fully symptomatic, but not at earlier stages.^33^ In the same study, *C9orf72* expansion carriers were found to have smaller putaminal volumes in asymptomatic and prodromal stages (1–6%) through to fully symptomatic stages (17%), while *MAPT* mutation carriers were only abnormal at a fully symptomatic stage (17%).

Including pathologically confirmed cases, an early investigation showed no volumetric differences in any brain region comparing tau and TDP-43 cases.^34^ However, in a more detailed study looking at pathological subtypes the group of patients with FUS pathology showed the most severe degree of caudate atrophy in comparison with controls (34% difference from controls),^15^ aligning with the evidence from multiple prior case series.^35^^,^^36^ Although with lesser severity, caudate atrophy was also seen across multiple pathologies e.g. Pick’s disease (23% difference from controls), corticobasal degeneration (CBD) (15%), TDP-43 type A (14%), frontotemporal dementia with parkinsonism linked to chromosome 17 (FTDP-17) (13%) and TDP-43 type C (11%). Furthermore, in this study, atrophy in the putamen was more marked than in the caudate in most groups (apart from FUS): Pick’s disease (38% difference from controls), FUS (33%), FTDP-17 (25%), CBD (24%), TDP-43 type A (25%) and TDP-43 type C (19%).

The ventral striatum, in the form of the nucleus accumbens, has been less studied than the dorsal region. However, atrophy in this region occurs in both bvFTD and svPPA, with volumes from 30% to 50% smaller than controls.^7^^,^^14^^,^^26^^,^^28^^,^^29^ Among the genetic forms, only symptomatic *MAPT* mutation carriers showed smaller volumes in the nucleus accumbens (11%).^33^ There may be asymmetry in the progression of atrophy. For example, in patients with TDP-43 type C pathology, atrophy of the left nucleus accumbens precedes the right.^37^ Current studies have not addressed whether the core or shell of the accumbens is more affected in different forms of FTD.

##### Pathology

Several studies have characterized *post mortem* striatal volume loss^38^ and histopathology.^39^^,^^40^ Consistent with neuroimaging evidence, the most severe striatal atrophy has been seen in cases with FUS pathology,^41^ especially in the caudate.

Studies focussing on genetic forms of FTD confirm the abnormalities of the basal ganglia, with prevalent involvement of the caudate in cases with *GRN* mutations,^41^ and severe neuronal loss and gliosis of the striatum along with TDP-43 inclusions.^42^ In FTDP-17, macroscopic atrophy is detectable at the intermediate stage in the caudate nucleus, whilst caudate and putaminal volume loss is evident in advanced illness.^43^

Patients with both tau and TDP-43 pathology show neuron loss, astrogliosis and focal microvacuolation in the ventral striatum, accompanied by tau or TDP-43 immunoreactive neuronal cytoplasmic inclusions and dystrophic neurites. In particular, svPPA patients with TDP-43 type C show abundant and focal neuronal cytoplasmic inclusions in the accumbens.^26^ In these patients with frontotemporal lobar degeneration (FTLD), the greatest amount of TDP-43 pathology is in the ventral striatum, followed by the putamen and the dorsal caudate.^44^

##### Symptomatology

Striatal degeneration determines diverse symptoms of FTD, directly and indirectly via the striatal projections to other regions. For example, striatal lesions are associated with decreased globus pallidus inhibition which leads to enhanced thalamic inhibition and reduced cortical activation. Striatal atrophy is therefore associated with disinhibition,^7^^,^^45^ binge eating^19^^,^^46^ and poor memory recall.^47^ Loss of the striatum innervation and atrophy can also result in akinesia and parkinsonism, which are reported in over half of the patients with FTD^48^ and a third of patients with right-temporal variant FTD.^39^ Parkinsonism has also been observed in nfvPPA, and linked to the progressive striatal atrophy and dopamine depletion in the putamen and caudate that characterize this syndrome.^49^

The role of the dorsal striatum and its connectivity with the frontal lobe is mirrored between FTD and other lesions to the dorsal fronto-striatal network, with executive dysfunction and behavioural impairment in FTD. In particular, dysexecutive syndromes are also associated with atrophy of the regions connected to the dorsolateral prefrontal cortex (e.g. antero-dorsal head of the caudate). On the other hand, neuropathology in the ventromedial head of the caudate and its connections to the orbitofrontal cortex leads to loss of socially appropriate behaviours, abnormal reward-seeking and disinhibited or impulsive behaviours.^19^ The nucleus accumbens has a key role in the representation of rewards associated with response options to stimuli, and it represents the outcome value of actions, weighting short and long-term consequences. Damage alters the representation of risks, for immediate versus delayed gratification.^23^ This explains why degeneration of this nucleus leads to impulsivity and disinhibition, typical of bvFTD.^50^ In bvFTD and svPPA, this may present with disinhibited sexual behaviours, repetitive or compulsive behaviours, abnormal eating behaviour and substance abuse.^26^^,^^51^^,^^52^ Degeneration of the nucleus accumbens or ventromedial caudate can also lead to apathy, as observed in all FTD syndromes, as a result of reduced motivation.^21^^,^^53–55^ Moreover, there is an association between reduced putamen volumes and severity of behavioural symptoms in FTD.^14^^,^^19^^,^^56^ Laterality effects may also be present but are less consistently reported. For example, overeating and sweet preference in bvFTD patients has been associated with selective right striatal degeneration.^19^^,^^46^

In genetic FTD, impairment of negative, as well as positive, outcome representations may account for the association of striatal atrophy with abnormal pain perception in *C9orf72*-associated FTD.^57^ Abnormal reinforcement learning as a result of striatal atrophy may also underpin psychotic symptoms, which is particularly common in *C9orf72*-associated FTD.^58^ In *GRN*- and *MAPT*-associated FTD, striatal atrophy is related to impaired social cognition.^59^

#### Globus pallidus

##### Anatomy

Another important nucleus of the basal ganglia is the globus pallidus, which has been closely associated with motor symptoms and signs, but which also mediate cognitive functions.^60^ As part of the ‘motor’ circuit, the ventrolateral globus pallidus receives input from the putamen and projects to the ventrolateral thalamic nucleus, which is linked to the supplementary motor cortex. The dorsomedial globus pallidus is connected to the anterior caudate and ventral anterior thalamic nucleus, while the lateral pallidus is connected to the dorsolateral caudate. The latter projects indirectly to the dorsolateral prefrontal cortex, creating a circuit linked to executive function and behavioural control.^20^ Ventral and antero-lateral regions of the pallidus are also connected to the nucleus accumbens, as part of the limbic system, mediating reward and thereby impulsive or inappropriate actions in FTD.

##### Neuroimaging

In bvFTD, the globus pallidus has been shown to be approximately 10% smaller than controls.^7^^,^^26^^,^^28^ The only study looking at genetic FTD across disease stages has found that fully symptomatic *MAPT* and *GRN* mutation carriers showed smaller volumes compared to controls (12–14%), while *C9orf72* expansion carriers showed reduced volumes (6%) even at a prodromal stage, reaching 16% volumetric difference when fully symptomatic.^33^ The relatively small size of the pallidus, and poor contrast to noise in many standard volumetric MRI sequences, means that its importance to FTD symptomatology may have been under-recognised from previous imaging studies.

##### Pathology

In a study on pathologically confirmed cases of FTLD, only few TDP-43 inclusions were found in the globus pallidus and ventral pallidus, compared to other basal ganglia structures.^44^ FUS-positive lesions were found in the globus pallidus in bvFTD with neuronal intermediate filament inclusion disease pathology.^61^ These were less frequent in bvFTD cases with atypical FTLD with ubiquitinated inclusions. A case study of a patient with bvFTD and TDP-43 type C revealed severe neuronal loss, gliosis and TDP-43 inclusions in the pallidus as for other regions of the basal ganglia.^62^ Cases with bvFTD and parkinsonism due to a mutation in *MAPT* showed tau positive inclusions in the pallidus, together with gliosis and neuronal loss.^63^

##### Symptomatology

The pallidus plays an important role in response inhibition, reducing thalamocortical output and consequently the activation in the motor cortex.^64^^,^^65^ Consequently, pallidus atrophy can be associated with motor perseveration and disinhibition, which are common symptoms in FTD.^66^^,^^67^ In particular, pallidus atrophy in these patients has been linked to the ‘applause sign’^67^ and poor performance at the Go/No-Go Task of inhibitory control.^68^ In addition, given the role of the ventral pallidus in the reward processing,^69^ its degeneration in FTD correlates with higher reward-seeking behaviours.^19^ In fact, clinical studies suggested that pallidal lesions can lead to apathy and anhedonia symptoms,^70^^,^^71^ which are prevalent across all FTD syndromes,^72–74^ and emerge even in presymptomatic mutation carriers.^75^^,^^76^

#### Other parts of the basal ganglia

##### Anatomy

The basal ganglia include the substantia nigra and subthalamic nucleus. The substantia nigra is connected via the subthalamic nucleus to the globus pallidus in the cortico-striato-pallido-thalamic-cortical circuits, regulating motor, reward and executive functions.^20^ The subthalamic nucleus is also connected to the amygdala, the orbitofrontal and inferior frontal cortex.^77^

##### Neuroimaging

Given the relatively small size of these nuclei, there are very few studies looking at volumetric differences in the substantia nigra and subthalamic nucleus in the FTD spectrum, and the main ones are related to parkinsonian syndromes. One study investigating the iron content in a cohort of bvFTD and PPA patients failed to find any difference in the substantia nigra,^78^ while a *post mortem* 7 T MRI study found a significant increase of iron deposition in the subthalamic nucleus of the FTLD-FUS, FTLD-TDP-43 and pure ALS groups, but not in the FTD-tau, while there was no difference in the substantia nigra from controls.^79^

##### Pathology

A case of bvFTD with confirmed TDP-43 type C showed severe neuronal loss and gliosis without TDP-43 inclusions in the subthalamic nucleus, while the substantia nigra was spared.^62^ FTDP-17 causes mild neuronal loss and gliosis of subthalamic nucleus and depigmentation of the substantia nigra.^63^ Overall, the subthalamic nucleus is typically atrophic with gliosis in the closely related disorder of PSP, while the substantia nigra shows pallor in most FTLD pathological forms.^80^

##### Symptomatology

Nigrostriatal neurons from the substantia nigra pars compacta regulate the subcortical–cortical loops for motor, oculomotor and cognitive control, through their terminations to the striatum. Neurodegeneration of this structure and reduced level of dopamine in the substantia nigra is typically associated with parkinsonian syndromes.^81^ Parkinsonism with rigidity and akinesia is commonly seen in bvFTD,^82^ in sporadic FTD and especially in FTD arising from mutations in *MAPT, GRN* and *C9orf72*. Specifically, in *GRN* mutation carriers, the parkinsonism correlates with the degeneration and neural inclusions in the substantia nigra.^83^ Pathology in the subthalamic nucleus and its connections is particularly associated with disinhibition,^64^^,^^65^ but can also affect emotion recognition and prosody decoding,^77^ all of which are commonly affected in bvFTD.

### Medial temporal lobe

#### Hippocampus

##### Anatomy

The hippocampus is a key component of the medial temporal lobe, involved in the formation of new memories and associated with learning and emotion.^84^^,^^85^ Specifically, the posterior hippocampus is involved in memory processing, through its connections with the medial and lateral parietal, medial prefrontal and posterior cingulate cortex, via a pathway involving the fornix projections to the mamillary bodies of the hypothalamus, anterior thalamic nucleus and anterior cingulate.^86^^,^^87^ The anterior part is instead more involved in emotion regulation, sensory–motor integration and goal-directed activity, due to the connections with the limbic structures (amygdala, nucleus accumbens, ventromedial prefrontal, orbitofrontal, anterolateral temporal, temporal pole, insula and cingulate cortex).^84^^,^^86^^,^^88^^,^^89^

The hippocampus is composed of different cytoarchitectonic subfields, mainly part of the allocortex, which have specialized functions and distinctive connections.^85^^,^^90^ Four main systems have been described^87^: an *episodic memory network* (originating in the subiculum and involving the anterior dorsal and lateral dorsal thalamic nuclei, mamillary bodies and retrosplenial cortex); an *emotional–social anterior network* (connecting the anterior CA1 and subiculum with the prefrontal cortex, amygdala and nucleus accumbens); a *sensory processing and integration system* (connecting CA1 and the subiculum with the parahippocampal cortex); and a *network for familiarity signalling and retrieval processing*, with extensive connection between the hippocampus, the prefrontal cortex and the thalamus.

The principal efferents of the hippocampus are the subiculum and CA1, except for the basal forebrain and nucleus accumbens, which are mainly connected with CA3.^87^ Further distinction and indirect pathways have been identified. The posterior part of the subiculum is connected to the lateral and medial parietal cortex, the frontal cortex and the striatum, while CA4 and the dentate gyrus are connected with the temporal and posterior cortex.^86^^,^^91^ The dorsal CA1 and subiculum are connected to the mamillary bodies and anterior thalamic nuclei, important for exploratory behaviour and spatial navigation, while their ventral regions are connected to the amygdala (central, lateral and basolateral nuclei), basal forebrain, medial hypothalamus and shell of the nucleus accumbens to regulate emotions.^89^ A resting-state functional study^91^ has found that among the hippocampal regions, CA1 was more strongly connected to the amygdala and occipital cortex, while CA2, CA3, CA4 and the dentate gyrus were more strongly connected to the left anterior cingulate, temporal and occipital cortex, while the subiculum to the angular, precuneus, posterior cingulate, frontal cortex and putamen.

##### Neuroimaging

Hippocampal atrophy has traditionally been described as particularly characteristic of Alzheimer’s disease, but many studies over the past 20 years have shown its involvement in some forms of FTD.^9^^,^^28^^,^^92^^,^^93^bvFTD have been reported to have 17–23% smaller hippocampus than controls.^7^^,^^28^^,^^29^ svPPA is associated with a characteristic pattern of asymmetrical atrophy of the anterior hippocampus (left greater than right), with usually 25–39% difference from controls on the left and 12–22% on the right^92–96^; and an annualized rate of atrophy of 0.14 on the left and 0.18 ml per year on the right.^97^ In contrast, studies generally report no significant hippocampal atrophy in those with nfvPPA.^92^

Looking at the genetic forms of FTD, the hippocampus is particularly atrophic in *MAPT* mutation carriers compared with *C9orf72* and *GRN* mutation carriers.^98–100^ Volume loss occurs around 15 years before expected onset in *MAPT* mutation carriers,^17^ with a faster annual rate of atrophy compared with other genetic forms of FTD.^31^^,^^101^

In patients with pathologically confirmed FTD, the hippocampus has been shown to be significantly smaller in Pick's disease (33% volume difference from controls) as well as in FTDP-17 (i.e. *MAPT* mutations: 43%) and TDP-43 type C cases (usually those with svPPA: 33%). Other pathologies have involvement to a lesser extent: FUS (31%), TDP-43 type A (23%) and CBD (14%).^15^

Hippocampal subfields have also been investigated in FTD. In svPPA, both the CA1 and subiculum regions were significantly smaller in svPPA than controls (27% and 24% volumetric difference, respectively).^96^ In a study of genetic FTD, a differential pattern of involvement was seen in the different groups: *MAPT* mutation carriers showed a 24–27% volumetric difference in the hippocampus proper (formed by the CA subfields), whilst *C9orf72* expansion carriers showed most atrophy in the dentate gyrus and CA1/4 (8–11%), and *GRN* mutation carriers were most affected in the subiculum and presubiculum (10–14%).^100^ In a larger study looking at different disease stages,^33^ all hippocampal regions were smaller than controls for fully symptomatic carriers for mutations in all three major genes. Differences were detected in several regions at asymptomatic and prodromal stages in both *MAPT* (the earliest in subiculum, presubiculum and tail) and *C9orf72* groups (the earliest in dentate gyrus, CA1/4 and presubiculum), and in the presubiculum (8%) in the prodromal stages of *GRN* mutation carriers.

##### Pathology

At *post mortem*, the hippocampus shows mild to severe neuronal loss, with 48% of cases showing hippocampal sclerosis (typically TDP-43 proteinopathies) and 64% showing the classic ubiquitinated inclusions in the dentate gyrus.^12^^,^^102^ In cases with confirmed TDP-43 pathology, the head of the hippocampus shows an average 57% atrophy in svPPA compared with controls, while bvFTD has 46% atrophy, more evenly distributed along the hippocampus.^103^ Pick’s bodies are consistently found in the granule cells of the dentate gyrus, the pyramidal cells of CA1 and the subiculum,^12^^,^^13^^,^^102^ whilst tau-positive grains and pre-tangles are found in the CA1 and dentate gyrus.^12^

Pathological studies in general are consistent with *in vivo* imaging of the genetic groups: tau deposition is extensively found in the hippocampus and other limbic structures in *MAPT* mutations^104^; dipeptide repeat proteins, together or without TDP-43 deposition, are found in the CA subregions in *C9orf72*; while TDP-43 accumulates in the hippocampus and the cortex in *GRN*.^105^

##### Symptomatology

The hippocampus is central to memory. Although significant episodic memory impairment is an exclusion criterion under current diagnostic criteria for bvFTD, improving the distinction from other causes of dementia, episodic memory can be affected in FTD. Indeed, several of Pick’s original cases had prominent memory symptoms, and one study reported amnesia in the initial clinical evaluation of 10% of pathologically confirmed cases.^106^ In bvFTD, memory deficits can co-occur with executive dysfunction, and involve both retrograde and anterograde memory performance. Similarly, svPPA patients can present episodic memory deficits, while nfvPPA patients generally show only mild autobiographical memory difficulties.^107^ Such episodic memory deficits in both bvFTD and svPPA are attributable in part to hippocampal dysfunction. Specifically, there is an association in bvFTD between hippocampal degeneration and deficits in memory recall and storage impairments,^47^^,^^108^ and episodic future thinking deficits.^109^ Hippocampal dysfunctions also relate to reduced mind wandering capacity^110^ and scene construction performance.^111^ However, despite the presence of hippocampal degeneration, FTD patients typically show preserved spatial navigation capacity in contrast to patients with Alzheimer’s disease and structural hippocampal lesions, suggesting that degeneration in the posterior parietal structures and other brain regions may mediate these deficits.^112^

Hippocampal deficits are relevant to more than memory domains in FTD, as this structure is involved in emotion modulation and evaluation of facial emotion.^113^ These are impaired not only in bvFTD but also in svPPA, especially right semantic dementia, and ‘temporal variant’ FTD.^114^ Atrophy in the anterior hippocampus in bvFTD and svPPA correlates with the typical symptoms of these two clinical groups: apathy and impaired social conduct in bvFTD, and anomia and impaired single word comprehension with preserved episodic memory in svPPA.^115^ Hippocampal volume is also reduced in FTD patients presenting with obsessive–compulsive behaviours.^116^ There are genetic influences on the hippocampal pathology and symptomatology. For example, severe medial temporal atrophy is seen in *MAPT* mutation carriers, with the greater involvement of the anterior and central regions of the hippocampus which form part of the limbic system: this is associated with the difficulties seen in these patients in regulating emotion and goal-directed behaviour.^31^

#### Amygdala

##### Anatomy

The amygdala is a limbic structure, composed of several subnuclei with different connections to the rest of the brain.^117^^,^^118^ The amygdala is involved in motivation, emotion, reward learning and in other cognitive functions (attention, perception and explicit memory).^117^

The nuclei of the amygdala are heterogeneous in composition, connections and roles.^117^ The lateral and basal amygdala are considered cortical regions, while the central and medial nuclei are considered ventral extensions of the striatum. The lateral nucleus is the ‘gatekeeper’ of the amygdala, as the major receiver of inputs from sensory and somatosensory systems, and important for processing of pain, fear learning and memory. The central nucleus is instead the most important output region, key for the control of motivation, emotional and behavioural responses, and connected to the brainstem, striatum, thalamus (mediodorsal, pulvinar and central nucleus), basal forebrain and lateral hypothalamus.^117^^,^^118^ Together with the central nucleus, the accessory basal, basal and paralaminar nuclei are considered the main component of the reward system within the amygdala, to motivate and reinforce behaviours.^117–121^

##### Neuroimaging

Amygdalar atrophy is common in bvFTD with prior studies reporting a 19–33% volume loss on the right and 22–41% on the left.^122^^,^^123^ Patients with svPPA show more atrophy than those with nfvPPA and bvFTD,^14^^,^^123^ with a strong asymmetry: 51–65% volume difference on the left (when left-predominant svPPA), and 33–54% on the right amygdala when compared to controls.^9^^,^^94^^,^^95^^,^^97^^,^^123^ Volume loss is smaller in bvFTD, around 10–19% smaller than controls in one study, with an annual atrophy rate of 4%.^7^^,^^27^^,^^28^

Studies of genetic FTD have shown that the amygdala is particularly affected in *MAPT* mutation carriers,^31^^,^^99^ a decade or more before the symptom onset.^17^ With recent developments in imaging technology, the amygdalar subnuclei are now measurable on MR imaging.^124^ Symptomatic *MAPT* mutation carriers showed smaller volumes particularly in the superficial and accessory basal regions (44%), which were 2–4% smaller even at an asymptomatic stage.^33^ Whilst *GRN* mutation carriers only showed smaller volumes than controls when fully symptomatic, *C9orf72* expansion carriers showed reduced volumes in all amygdalar regions even at the asymptomatic and prodromal stages, with the main reduction being in superior-medial regions.^33^

In pathologically confirmed cohorts, amygdalar atrophy is most marked in cases with FTDP-17 (*MAPT* mutation carriers) and TDP-43 type C (usually svPPA),^15^^,^^125^^,^^126^ with 50% smaller volume than controls.^15^ When looking at the evolution of brain atrophy in svPPA with and without confirmed TDP-43 type C pathology, the amygdala was found to be affected at the very early stages on both sides.^37^^,^^126^^,^^127^ However, there is also significant involvement in those with Pick’s disease (45% difference from controls), more so than those with FUS (37%), TDP-43 type A (25%) or CBD (20%) pathology.^15^^,^^126^ For the amygdalar subregions, a recent study of pathologically confirmed FTD cases reported a differential involvement with the medial subnuclei (particularly the superficial, accessory basal and basal/paralaminar subnuclei) being more affected than the lateral subnuclei.^126^

##### Pathology

Neuropathological studies have shown severe amygdalar volume loss of 52% in FTD.^128^ One study on TDP-43 pathology showed inclusions in the basolateral nucleus of the amygdala in the earliest stage of the disease.^129^

##### Symptomatology

Amygdala is a key area in the reward and punishment system. The core behavioural features and symptoms of FTD (i.e. lack of insight, impaired personal and social conduct, disinhibition) are consistent with the loss of function of the amygdala, and are regularly seen with all types of FTD-related pathology.^12^^,^^13^ The subnuclei most affected in FTD are connected to other limbic regions. Across all FTD variants, atrophy of the amygdalar subnuclei relates to a wide range of behavioural and neuropsychiatric scales,^14^ either directly or through the deficits in the reward and emotional processing caused by bvFTD and svPPA.^130^^,^^131^ The medial nuclei are likely related to the development of symptoms associated with abnormal reward and emotional processing, relative to the salience and limbic networks^132^^,^^133^: indeed the amygdala plays a role in evaluating the motivation and emotional context of a given stimulus, and feedbacks the information to the brainstem to control emotional reaction, and to the striatum to control actions.^117^

Atrophy and hypometabolism of the amygdala relate to deficits in the emotion processing and recognition, and social interaction insight.^134–138^ It is also linked to impaired comprehension of intentionality,^139^ and insensitivity to negative stimuli.^140^ Some reports indicate right sided associations^134^^,^^135^^,^^137^^,^^138^ while others reveal left sided associations,^137^^,^^141^ especially involving the superficial and basolateral nuclei. Deficits in social cognition, interoceptive accuracy or emotion comprehension were found to be related to amygdalar atrophy on the right hemisphere or bilaterally in svPPA^138^^,^^142^ and the temporal variant of FTD.^114^ In patients with the ‘right temporal variant of FTD’, deficits in facial expression recognition, reduced empathy and emotional reaction are commonly observed.^114^ Emotion recognition deficits correlate with atrophy in the left amygdala in patients with nfvPPA.^143^

#### Basal forebrain

##### Anatomy

The basal forebrain is a collection of cholinergic nuclei, including the diagonal band of Broca, the medial septal nucleus and the nucleus basalis of Meynert.^144^ Via cholinergic pathways linking the cortex and limbic system, they are essential for different cognitive processes, including memory, learning and attention.^145^^,^^146^

##### Neuroimaging

Basal forebrain volume is reduced in both svPPA and nfvPPA as compared to controls, mainly in the posterior part of the nucleus basalis.^147–149^ Patients with bvFTD and svPPA were reported to have significantly lower volumes than controls (9–10%) and nfvPPA (4–5%), with FTD-ALS and all PPA variants also having lower volumes than controls (5–9%).^150^ Among genetic cases, only fully symptomatic *MAPT* mutation carriers showed significantly smaller basal forebrain volumes than controls (15–18%) and both *GRN* and *C9orf72* groups (14–17%).^33^^,^^150^ In the same study,^150^ pathologically confirmed cases with tau showed the smallest basal forebrain volumes (mainly driven by FTDP-17 and Pick's disease) than controls (10%), while among the TDP-43 proteinopathies, the lower volumes were driven by those with TDP-43 type C pathology.

##### Pathology

In one study, TDP-43 inclusions have been found in the basal forebrain (including the diagonal band of Broca, nucleus basalis of Meynert and substantia innominata).^44^ Patients with PPA showed a severe reduction in the cholinergic neurons in the nucleus basalis of Meynert and nucleus subputaminalis.^151^

##### Symptomatology

Atrophy and pathology of the basal forebrain can lead to diverse symptoms in FTD, but a unifying framework for these effects is outstanding. The cholinergic system plays a key role in cognitive processing, and the cholinergic dysfunction seen in bvFTD and PPA arise from the degeneration of the nucleus basalis. For example, language impairment in PPA has been partially attributed to the cholinergic deficits from the basal forebrain pathology.^147^^,^^148^^,^^152^^,^^153^ A role of the basal forebrain in social cognition and attachment has been proposed, given the density of receptors for oxytocin and vasopressin.^154^ This accords with the association between basal forebrain hypometabolism with abnormal prosocial sentiments in bvFTD (i.e. pity and guilt).^155^ Despite these associations, cholinergic dysfunction seems to be more marked in CBS and PSP than other FTD syndromes, which may in part explain why cholinesterase inhibitors have not proven effective to improve cognitive function and behavioural symptoms in bvFTD and PPA.^156^^,^^157^

### Thalamus, hypothalamus and habenula

#### Thalamus

##### Anatomy

The thalamus is the relay station of the brain, and it is connected to the majority of other regions. It is composed of several nuclei, each of them with specific connections and functional specialization.^158^ While the anterior, lateral, ventro-anterior and medio-dorsal nuclei are considered limbic structures, the ventrolateral and ventromedial are considered motor, and the latero-posterior, ventro-posterior lateral, midline and intralaminar are considered associative and somatosensory (reviewed in Bocchetta et al.^159^). The lateral and medial geniculate nuclei have specific sensory functions in the visual and auditory system, while the pulvinar plays a role in the intramodality integration of somatosensory and visual information, and in the presence of affective and psychotic symptoms, including hallucinations.

##### Neuroimaging

Thalamic atrophy is a common feature across all clinical, genetic and pathological forms of FTD,^17^^,^^160–162^ and occurs even in the early clinical stages.^8^ bvFTD and nfvPPA show bilateral atrophy in the anterior and posterior thalamus.^14^^,^^16^^,^^28^ Both Pick’s disease and TDP-43 type A groups showed asymmetric volume loss in the thalamus.^15^

Among the genetic groups, the *C9orf72* expansion carriers have been considered to be the ones where the thalamus was particularly affected, even presymptomatically^17^^,^^163^ when considering either those between 20 and 40 years of age^164^ or those without any clinical symptoms.^33^ However, whilst there is more widespread involvement of the thalamus later, it seems that the earliest change is in the pulvinar nucleus,^165–167^ even at a presymptomatic stage^168^; this region tends to be less affected in all other forms of FTD.^159^ In a detailed study of thalamic subnuclei, the medial dorsal was affected across all clinical, genetic and pathological FTD subgroups.^159^ Changes in the thalamic regions only become visible at the fully symptomatic stages in both *GRN* and *MAPT* mutation carriers, with atrophy mainly localized in the medial dorsal, midline and laterodorsal nuclei (22–31%), while the lateral geniculate nucleus was spared in both groups, but atrophic in *C9orf72*.^33^

##### Pathology

*Post mortem* volume analysis of svPPA confirmed 27% loss of volume in the anterior thalamus^103^ and 34% reduction for bvFTD, with confirmed TDP-43 pathology. From a subnuclei point of view, pathological studies have shown a marked involvement of the medial dorsal nucleus, with neuronal loss, gliosis and astrocytosis in bvFTD.^169^ However, pathological hallmarks of FTD are not evenly distributed in the thalamus. For example, TDP-43 is mainly found in the medial nuclei of thalamus (including anterior, lateral dorsal and dorsomedial nuclei), in the periventricular thalamic neurons, while few inclusions are found in the lateral nuclei of the thalamus.^44^

##### Symptomatology

The heterogeneity of thalamic nuclei and their position within parallel cortico-striato-thalamo-cortical loops for cognition and motor control means that the thalamic pathology in FTD gives rise to diverse symptoms and signs. However, there is ongoing work looking at the specificity of the thalamic origin of the FTD symptoms, trying to accurately localize the thalamic nucleus involved for each clinical phenotypes and genetic forms. It is also challenging to dissociate the direct effects of pathology of the thalamic nuclei, from changes in their afferent and efferent connections,^19^ and degeneration of the cortical projections of each nucleus.

The symptomatology of thalamic changes in FTD follows the functional anatomical circuits discussed above. For example, in *C9orf72* expansion carriers the pulvinar pathology is consistent with impairment of limbic functions and intramodality integration of sensory information,^158^ including altered processing of pain, hallucinations, affective and psychotic manifestations of FTD.^170–172^ In bvFTD, atrophy in the pulvinar also relates to lower prosocial giving,^173^ consistent with an integrative role in social, affective and reward processing. In contrast, pathology in the medial dorsal nucleus in different variants of FTD affects connectivity with widespread brain regions including orbital, lateral and dorsal prefrontal cortex, and other limbic regions. This can exacerbate emotional and executive impairments over and above the cortical pathology. Damage in the thalamic regions forming part of the anterior cingulate circuits have been associated with changes in apathy and memory.^169^ As part of the Papez circuit, thalamic degeneration may increase memory deficits, where for example atrophy in the thalamus and fornix has been reported to be associated with severity of amnesia in bvFTD.^162^

#### Hypothalamus

##### Anatomy

The hypothalamus plays an important role in food intake, reward and perception of satiety. It also regulates the homeostasis of neuroendocrine, behavioural, and autonomic processes, including circadian rhythm, stress response, sexual and defensive behaviours and thermoregulation.^174^^,^^175^ It is composed of a number of different subnuclei and is highly interconnected with other parts of the central nervous system, particularly the brainstem, limbic system and cortex. Besides axonal connections, the hypothalamus contains neuropeptide-expressing neurons and neuropeptide receptors, and it engages with the pituitary gland to release hormones into the bloodstream.^175^^,^^176^ The nuclei involved in the food intake are mainly the lateral hypothalamus, and the arcuate, dorsomedial and paraventricular nuclei.^175^

##### Neuroimaging

In a study of eighteen people with bvFTD, hypothalamic volume was reduced 17% compared with controls, with the main differences localized to the superior parts of the anterior and tuberal regions and the posterior region, which regulate appetite.^177^ Another study has confirmed atrophy of the hypothalamus in bvFTD, particularly in its posterior portion, but not in svPPA.^178^

In a small study which included those with genetic FTD, atrophy was significantly more severe than controls in *MAPT* mutation carriers (in superior and posterior areas), but not in those with *C9orf72* expansions. In particular, the posterior part of the hypothalamus was the most affected area, including the mamillary bodies, which are connected to the amygdala and hippocampus, both structures known to be particularly atrophic in *MAPT* mutation carriers.^177^ In a larger study across disease stages,^33^ hypothalamic volumes were smaller in fully symptomatic mutation carriers (except for the inferior tubular regions in *C9orf72* and *GRN*), with *MAPT* symptomatic carriers showing up to 29% smaller volumes in the posterior and anterior regions. However, the only group showing smaller volumes before symptom onset was *C9orf72*, especially in the superior anterior and tuberal regions.

##### Pathology

Multiple pathologies have shown involvement of the hypothalamus. For example, one study showed a volume reduction of 41% in bvFTD and TDP-43 pathology cases when compared to controls.^103^ In a *post mortem* study of 19 cases with TDP-43 pathology, inclusions were found in the lateral hypothalamic area, tuberomammillary nucleus, lateral tuberal nucleus, preoptic area, ventromedial and dorsomedial nuclear groups, and in the posterior hypothalamic area. No inclusions were seen in other hypothalamic regions.^44^ The hypothalamic lateral tuberal nucleus was also shown to be severely affected in Pick’s disease.^12^ Cases with tau pathology and Pick’s disease showed more abnormal protein deposition than cases with TDP-43 type B, with this latter showing more severe posterior hypothalamic atrophy than the tau group.^179^

##### Symptomatology

The key role of the hypothalamus in appetite, food-reward and the perception of satiety is reflected in the symptoms associated with its degeneration in FTD. Abnormal eating behaviours are present in up to 60% of patients with FTD, and particularly prominent in patients with bvFTD and svPPA.^180^ Hyper-orality and sweet tooth are diagnostic criteria for bvFTD,^1^ but the specific symptoms vary widely between individuals. bvFTD can present with complex eating behaviours, from overeating to sweet craving, to obsessions for specific foods.^181^ Alteration in eating behaviours may be driven by hypothalamic pathology via multiple processes, over and above cortical and striatal mediation of poor impulse control and environmental dependency. Posterior and whole hypothalamic atrophy has been shown to correlate with abnormal eating behaviours.^178^^,^^182^ Specifically, feeding behaviour alterations are related to localized degeneration in the lateral hypothalamic nuclei, and the arcuate and paraventricular nuclei.^183^ In contrast, lesions in posterior hypothalamus contribute to autonomic dysfunction and altered satiety responses.^184^

Structural and functional alterations of the hypothalamus are associated with autonomic deficits in bvFTD, such as lower baseline skin conductance levels.^185^ Cardiac, urinary and thermoregulatory dysfunctions have been reported in patients with FTD,^186^^,^^187^ as described in the original diagnostic criteria.^188^ Sleep disturbances are also prevalent in FTD,^189^ and might be associated with hypothalamic degeneration and the loss of its connections with the frontotemporal cortex.^190^ The direct sleep disturbances from FTD need to be separated from indirect effects of physical disability, motor deficits, poor sleep hygiene arising from altered lifestyle, and iatrogenic pharmacological impairments. Nonetheless, sleep disturbance as a result of FTD itself is common, including either hypersomnolence or insomnia. These can be refractory to treatment, arising from degeneration of central thalamic or hypothalamic regulators of circadian rhythms.

#### Habenula

##### Anatomy

The habenula is a small but key nucleus within the reward network.^191^ It integrates information from the other limbic structures and basal ganglia to generate goal-directed behaviours, by processing and balancing reward and adversity.^192^^,^^193^ The lateral habenula is connected to the lateral hypothalamic and lateral preoptic areas, basal forebrain, ventral pallidus, amygdala, substantia nigra and brainstem.^192^ It also receives input from the anterior insula, anterior cingulate and ventral frontal pole.^193^ The medial habenula is connected to the basal forebrain and midbrain.^193^

##### Neuroimaging

Only one small study has reported the habenula changes in FTD, showing a 29% lower volume in bvFTD compared with controls.^194^ Other studies have not reported this structure in FTD, perhaps due to its small size and the lack of an automated method which currently makes its quantification unfeasible in large cohorts.

##### Pathology

There are currently no studies reporting the presence of pathology or neurodegeneration in the habenula in FTD.

##### Symptomatology

The habenula mediates the processing of negative and aversive information, and suppresses actions when it is anticipated that these will not produce a reward or avoid a negative feedback.^193^^,^^195^ The habenula is activated by negative feedback.^196^ Given this function, neurodegeneration in the habenula can lead to perseveration (due to inconsistence use of negative feedback) or disinhibition and impulsivity (due to inability to avoid an action),^192^ and to the abnormal reward behaviours often seen in bvFTD patients. In addition, in animal studies, the lateral habenula and its connections with prefrontal regions have been reported as implicated in working memory and other executive functions,^197–199^ which are characteristically impaired in patients with bvFTD.^1^ The role of habenula in these functions is also supported by its modulatory role on the activity of the dopaminergic system.^200^^,^^201^ However, further work is required to establish the specific role of habenula dysfunction as a direct cause of behavioural change in FTD.

### Brainstem and cerebellum

#### Brainstem

##### Anatomy

The brainstem is divided into the midbrain, pons and medulla oblongata. The midbrain is associated with vision, hearing, sleep and motor control, and it also forms part of a network that regulates emotion perception with the thalamus and amygdala.^202^ The pons is connected to both the cerebrum and the cerebellum, via the cerebellar peduncles, and it is associated with respiration and facial expression. The medulla connects the cerebrum to the spinal cord, and regulates cardiac and respiratory functions, reflexes and integrative functions, such as consciousness, emotional processing, pain and motivation.^203^

##### Neuroimaging

Few imaging studies have focussed on the brainstem in FTD, perhaps because of the exclusion of the brainstem from early imaging atlases of grey matter. However, brainstem changes are typical of other tauopathies, and in particular CBS and PSP, with PSP showing marked atrophy in the midbrain and superior cerebellar peduncle.^204–206^ As PSP often overlaps clinically with nfvPPA and bvFTD,^3^^,^^207^ it is not surprising that patients with these overlapping syndromes present with brainstem involvement in addition to the typical cortical pattern of bvFTD and PPA.^208^ A study of 22 FTLD patients (5 of whom also met criteria for ALS) reported 10% smaller volumes than controls in the brainstem, including midbrain, pontine tegmentum, superior and inferior colliculi.^161^ In a diffusion imaging study, bvFTD, bvFTD with ALS, nfvPPA, and PSP patients showed abnormal measures in the brainstem, while in svPPA the brainstem was spared.^209^ This also suggested that patients with probable tau pathology (like nfvPPA and PSP) showed abnormal changes in the brainstem, superior and inferior cerebellar peduncles more than those with probable TDP-43 pathology (svPPA and bvFTD with ALS). However, the brainstem, and specifically the pons, has been found to be atrophic in *GRN* mutation carriers, who typically show TDP-43 pathology.^31^ This result was confirmed by a recent large study on genetic FTD,^33^ which reported 5–8% smaller volumes of the superior cerebellar peduncle, midbrain and pons in *GRN* mutation carriers, 9% smaller midbrain volumes in *MAPT* mutation carriers, but no difference in *C9orf72* expansion carriers, nor in any presymptomatic carriers.

##### Pathology

TDP-43 pathology and neuronal loss has been found previously in several nuclei of the midbrain and pons in cases with bvFTD, bvFTD and ALS, and PPA variants.^210^^,^^211^

##### Symptomatology

Despite the name ‘frontotemporal dementia’, brainstem pathology is commonly associated with functional impairment in FTD. Functional networks responding to salient events and enabling adaptive behaviour include brainstem nuclei, and they are impaired in FTD.^212^ The salience network is active in response to stimuli that are emotionally significant.^132^ In this way, brainstem degeneration contributes to some of the deficits in social cognition and emotion processing attributed to cortical pathology in FTD. In addition, the thalamus–amygdala axis for emotion and social perception is moderated by brainstem projections,^202^ and especially by the midbrain, which is affected in *MAPT* mutation carriers. The brainstem reticular activating system and its projections to the cerebrum are critical for arousal, and its neurodegeneration is associated with apathy in FTD.^74^

Beyond behavioural symptoms, the clinical overlap between FTD, PSP and other forms of parkinsonism also consists in motor symptoms, which are underpinned by brainstem degeneration, as identified by *post mortem* and imaging studies.^74^^,^^82^^,^^207^ These symptoms include impairment of oculomotor control by the superior colliculus in the midbrain tectum.^213^ Atrophy of the tectum occurs in FTD,^161^ explaining saccade abnormalities in these patients with and without PSP-aetiology.^214^ In particular, in PSP this manifests in slow and hypometric vertical saccades, and later a vertical gaze palsy.

Brainstem nuclei are the main sources of the principal modulatory neurotransmitter systems, including serotonergic, dopaminergic and noradrenergic innervation of the forebrain. FTD affects the serotonergic projections from the raphe nuclei, the dopaminergic projections from the ventral tegmental area and the noradrenergic projections from the locus coeruleus.^81^ Changes to such fundamental distributed systems are expected to have widespread consequences on cognition and behaviour. Indeed, serotonin dysfunction in FTD is confirmed by reduced transmission and postsynaptic receptor density, and relates to behavioural changes, such as aggression, impulsivity and increased appetite.^215–216^ In many patients, FTD is associated with depletion of nigrostriatal dopamine projections, loss of pre-synaptic dopaminergic neurons and altered dopamine receptor binding in the striatum. This leads to cognitive change, motor parkinsonism and vulnerability to iatrogenic extra-pyramidal symptoms.^82^^,^^217^ Impairment in the noradrenergic system from degeneration in the locus coeruleus is likely to contribute to the dysregulation of attention, memory and decision-making, although specific associations in FTD are yet to be established as they have been for PSP.^218–220^

#### Cerebellum

##### Anatomy

Traditionally, cerebellar function has been associated only with the coordination of movement, but recent studies have found that the cerebellum is important in cognitive and emotional processing.^221^^,^^222^ The cerebellum has several connections with key areas involved in FTD, in particular to the prefrontal cortex via the thalamus,^223^^,^^224^ and to the limbic system via a direct cerebello-limbic pathway.^225–227^ More specifically, the superior–posterior cortex (lobule VI, VIIa-Crus I, VIIa-Crus II, VIIIb), connected to the ventrolateral and ventro-anterior thalamus to the prefrontal cortex, has been associated with cognitive processing (executive functions, language, attention) and social cognition.^221^^,^^222^^,^^224^ The vermis is instead also called the ‘limbic cerebellum’, as it plays a role in the modulation of emotional and social behaviours.^221^^,^^225–227^ The anterior cerebellum is instead the area linked with motor/sensorimotor functions.^221^ The deep cerebellar nuclei (dentate, interposed and fastigial nuclei) receive intrinsic inputs from the cerebellar cortex to be sent to the other cortical regions via the ventro-anterior and ventrolateral thalamic nuclei.^221^

##### Neuroimaging

Differential involvement of the cerebellum has been shown in FTD, as also highlighted in a recent metanalysis of neuroimaging studies.^228^ Overall, in bvFTD changes were found in the Crus bilaterally, in the left lobule VI, in the right lobules VIIb and VIIIb, and part of the vermis.^229^^,^^230^ In svPPA, changes were asymmetric, and mainly located in the left Crus I and lobule VI,^228^ but also in the left lobules IV–V.^229^ Cerebellar atrophy was also observed in nfvPPA, localized bilaterally in the lobules VI, right Crus I and VIIb.^229^^,^^230^

The involvement of the cerebellum in *C9orf72* expansion carriers has been well characterized,^17^^,^^99^^,^^231^^,^^232^ with the lobule VIIa-Crus I and VIIa-Crus II in the superior–posterior region of the cerebellum particularly involved, even at the earliest presymptomatic stages.^33^^,^^233^ A small cohort of symptomatic mutation carriers^233^ found the cerebellum to be relatively spared in those with *GRN* mutations, and localized to the vermis in *MAPT* mutation carriers, the ‘limbic cerebellum’ involved in the modulation of emotions and social behaviours, as already mentioned.^221^^,^^225^ However, a larger cohort using the same methods did not confirm such differences in the *MAPT* group, but reported 8–13% smaller volumes in lobules VIIa-Crus II, VIIb and VIIIa in fully symptomatic *GRN* mutation carriers.^33^

##### Pathology

Interestingly, dipeptide repeat proteins, the characteristic pathology of *C9orf72* expansion carriers, are found throughout the cerebellum, in case with or without ALS phenotype.^5^^,^^232^^,^^234–236^  *Post mortem* examination of two siblings with bvFTD showed massive abnormal tau deposition in astrocytes in the cerebellum.^237^

##### Symptomatology

The cerebellum has long been associated with motor control, and its lesions with ataxia. Although uncommon, ataxia has been described in patients with *C9orf72* expansions.^238^^,^^239^

Beyond motor control and movement, cerebellar functions extend to all areas of cognition, including affective, social and executive domains. Its role in cognitive and emotional processing in FTD is now emerging. For example, in bvFTD, Tan et al.^240^ found an association between lobules V and VII (Crus I) and memory, language, executive and emotion deficits, together with an association between the vermis and memory and language dysfunction. Areas of cerebellar atrophy were linked with attention and working memory in bvFTD, visuospatial function in svPPA, and language-motor function in nfvPPA.^230^ Atrophy in the Crus and lobule VI was commonly associated with cognitive deficits in all FTD phenotypes, and in the Crus I and Crus II were associated with both behavioural disruption and cognitive dysfunctions.^228^

Changes in cerebellar connections have been linked with loss of episodic memory, attention, working memory, visuospatial, executive function and emotion in bvFTD; with working memory, language and emotion in svPPA; and with attention, language, executive function, working memory, visuospatial and emotion in nfvPPA.^241^ Altered emotion processing and motivation have been described in patients with cerebellar damage,^240^^,^^242^^,^^243^ and found related to cerebellar degeneration and disconnection in all FTD variants.^240^^,^^241^ Cerebellar degeneration also correlates with eating behaviours in both bvFTD and svPPA,^244^ and with decision making and theory of mind in bvFTD.^245–247^ In *C9orf72*, structural changes in the cerebello-thalamic-cortical network are seen early presymptomatically, and by the time *C9orf72* expansion carriers reach the symptomatic stage, they have disturbances of body schema and related neuropsychiatric symptoms related to cerebellar disease.^248^

## Conclusion

**Figure 2:**
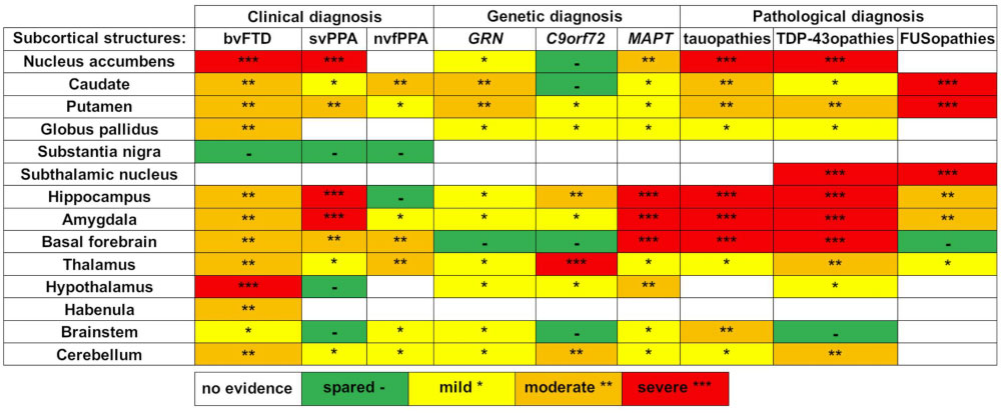
Overview of the involvement of the subcortical structures in the clinical, genetic and pathological groups of FTD.

Existing studies reveal extensive involvement of subcortical structures in the clinical, genetic and pathological forms of FTD. As summarized in Fig. 2, there is a complex differential pattern of atrophy in the different structures across the FTD spectrum. In general, bvFTD is associated with multiple regions of the reward network, including the nucleus accumbens, amygdala, hypothalamus and habenula. More specifically, by genetic and pathological group, the limbic structures (such as the amygdala, the hypothalamus, the posterior hypothalamus and the nucleus accumbens) are mainly affected in those with *MAPT* mutations as well as Pick’s disease, while the basal ganglia are mainly involved in those with *GRN* mutations and FUS pathology. *C9orf72* expansion carriers have a particular involvement in the pulvinar nucleus of the thalamus and the cerebellum, forming part of a cerebello-thalamic-cortical network related to neuropsychiatric symptoms in this group. Further studies are needed to explore and fully understand the role of these nuclei in all the forms of FTD, and in particular how their place within wider networks is lost as connections are broken down with disease progression.

The studies included in this review differ in a number of characteristics, including the imaging techniques used (manual or automated segmentation, volumetric region of interest or voxel-based morphometry), the sample size, the inclusion criteria for patients, the quality of MR images and the covariates included in the comparisons (disease duration, severity of symptoms, global atrophy). Therefore, results are not always directly comparable, or amenable to meta-analysis. Collaborative studies investigating multiple structures at the same time on large cohorts will be better able to provide a clearer picture of subcortical changes in FTD, including disease progression and variants. Adequately powered longitudinal studies, including sample size estimates, are essential to understand the variability of subcortical structures, especially small but critical structures, such as the habenula and subthalamic nucleus. These are difficult to identify using automated methods or from clinical-grade images. The anatomical definition of the boundaries of some of these structures is also a relevant source of heterogeneity, not only across neuroimaging studies, but also *post mortem* investigations. Initiatives like the harmonization of hippocampal subfields (www.hippocampalsubfields.com Accessed on 21 July 2021)^249^ are underway and will provide relevant resources to accurately address sources of variability. These investigations will be fundamental to develop MRI markers that include subcortical regions that are reproducible across studies, and for single subject assessments for stratification and monitoring in clinical trials.

With this review, we draw attention to the important role that the subcortical structures play in the spectrum of FTD, which has often been overlooked in the past. These regions are affected differently across the FTD disorders, and show clear early changes in the disease process.

## Funding

The Dementia Research Centre is supported by Alzheimer's Research UK, Brain Research Trust and The Wolfson Foundation. This work was supported by the National Institute for Health Research (NIHR) Queen Square Dementia Biomedical Research Unit, the NIHR UCL/H Biomedical Research Centre and the Leonard Wolfson Experimental Neurology Centre (LWENC) Clinical Research Facility as well as an Alzheimer's Society grant (AS-PG-16-007). MB is supported by a Fellowship award from the Alzheimer’s Society, UK (AS-JF-19a-004-517). MB’s work is also supported by the UK Dementia Research Institute which receives its funding from DRI Ltd, funded by the UK Medical Research Council (MRC), Alzheimer’s Society and Alzheimer’s Research UK. JDR is supported by an MRC Clinician Scientist Fellowship (MR/M008525/1) and has received funding from the NIHR Rare Disease Translational Research Collaboration (BRC149/NS/MH). JBR and MM were supported by the Cambridge University Centre for Parkinson-Plus, the Medical Research Council (SUAG/051 G101400) and the NIHR Cambridge Biomedical Research Centre (BRC-1215-20014). The views expressed are those of the authors and not necessarily those of the NIHR or the Department of Health and Social Care.

## Competing interests

The authors report no competing interests.

## Data availability

Data sharing is not applicable to this review article as no new data were generated or analysed in this study. Source study data may be available from the authors cited.

## Abbreviations

ALS =: amyotrophic lateral sclerosis
bvFTD =: behavioural variant frontotemporal dementia
CA =: cornu ammonis
CBD =: corticobasal degeneration
CBS =: corticobasal syndrome
*C9orf72* =: chromosome 9 open reading frame 72
FTD =: frontotemporal dementia
FTDP-17 =: frontotemporal dementia with parkinsonism linked to chromosome 17
FTLD =: frontotemporal lobar degeneration
FUS =: fused-in-sarcoma
*GRN* =: progranulin
lvPPA =: logopenic variant of primary progressive aphasia
*MAPT* =: microtubule-associated protein tau
nfvPPA =: non-fluent variant of primary progressive aphasia
PPA =: primary progressive aphasia
PSP =: progressive supranuclear palsy
svPPA =: semantic variant of primary progressive aphasia
TDP-43 =: TAR DNA-binding protein 43
